# Online Peer-to-Peer Support for Young People With Mental Health Problems: A Systematic Review

**DOI:** 10.2196/mental.4418

**Published:** 2015-05-19

**Authors:** Kathina Ali, Louise Farrer, Amelia Gulliver, Kathleen M Griffiths

**Affiliations:** ^1^ National Institute for Mental Health Research The Australian National University Canberra Australia

**Keywords:** mental health, Internet, young people, peer-to-peer support, Internet support groups, technology, systematic reviews

## Abstract

**Background:**

Adolescence and early adulthood are critical periods for the development of mental disorders. Online peer-to-peer communication is popular among young people and may improve mental health by providing social support. Previous systematic reviews have targeted Internet support groups for adults with mental health problems, including depression. However, there have been no systematic reviews examining the effectiveness of online peer-to-peer support in improving the mental health of adolescents and young adults.

**Objective:**

The aim of this review was to systematically identify available evidence for the effectiveness of online peer-to peer support for young people with mental health problems.

**Methods:**

The PubMed, PsycInfo, and Cochrane databases were searched using keywords and Medical Subject Headings (MeSH) terms. Retrieved abstracts (n=3934) were double screened and coded. Studies were included if they (1) investigated an online peer-to-peer interaction, (2) the interaction discussed topics related to mental health, (3) the age range of the sample was between 12 to 25 years, and (4) the study evaluated the effectiveness of the peer-to-peer interaction.

**Results:**

Six studies satisfied the inclusion criteria for the current review. The studies targeted a range of mental health problems including depression and anxiety (n=2), general psychological problems (n=1), eating disorders (n=1), and substance use (tobacco) (n=2). The majority of studies investigated Internet support groups (n=4), and the remaining studies focused on virtual reality chat sessions (n=2). In almost all studies (n=5), the peer support intervention was moderated by health professionals, researchers or consumers. Studies employed a range of study designs including randomized controlled trials (n=3), pre-post studies (n=2) and one randomized trial. Overall, two of the randomized controlled trials were associated with a significant positive outcome in comparison to the control group at post-intervention. In the remaining four studies, peer-to-peer support was not found to be effective.

**Conclusions:**

This systematic review identified an overall lack of high-quality studies examining online peer-to-peer support for young people. Given that peer support is frequently used as an adjunct to Internet interventions for a variety of mental health conditions, there is an urgent need to determine the effectiveness of peer support alone as an active intervention.

##  Introduction

 The mental health and well-being of young people is a major public health concern [1]. Findings suggest that one in four young people aged 16 to 25 years have experienced at least one mental health problem during their lifetime [2]. Although young people have one of the highest prevalence rates for mental health problems, their needs are often unmet and access to mental health services is limited [1]. Previous research has identified various barriers to treatment including concerns about confidentiality, lack of knowledge of resources, cost, and inaccessibility of services [3]. More broadly, young people are reluctant to seek professional face-to-face help for their problems [4].

It has been reported that a growing number of young people use the Internet to seek help and information regarding their mental health [5], and 93% of young people report being online on a regular basis [6]. Furthermore, online interventions might be of particular interest for this age group due to the high levels of anonymity, easy access independent of time and location, cost-effectiveness for large populations, and the potential of such interventions to be perceived as less stigmatizing [7-10]. These advantages could potentially overcome some of the barriers young people face when seeking help for mental health problems.

Peer-to-peer support is a promising aspect of online mental health interventions. Prior research estimated that millions of people access online support groups daily [11]. Peer-to-peer support enables young people to connect with others, share experiences, seek and provide information, advice, and emotional support, and is often delivered as part of complex multi-component online interventions [8]. Research has shown that a majority of young people use the Internet to connect with others [12] suggesting that online peer-to-peer support could be a powerful tool to help reduce stigma and increase help-seeking for mental health problems.

A wide variety of online peer support platforms exist, including asynchronous (Internet support groups (ISGs)/discussion groups/bulletin boards/forums) and synchronous (chatrooms, virtual reality environments) formats. Available evidence suggests that online peer-to-peer support interventions might be beneficial for users [11]. Research has shown that users of online forums improve their coping strategies, both in social interaction and with regard to their health condition [13]. Peer support may also increase supportive communication [14] and emotional well-being [15]. Previous studies showed that greater participant involvement in an online forum was associated with lower levels of emotional distress among adolescents [16]. A meta-analysis of peer support interventions for depression found evidence that peer support leads to improvements in depressive symptoms relative to usual care [17]. A systematic review of Internet support groups for a wide variety of health conditions also found positive effects for depressive symptoms [18].

Although reviews have evaluated the effectiveness of face-to-face and online peer support networks in adults with a variety of health conditions, none have directly examined peer-to-peer support for mental health problems in young people. A recent systematic review examined online and social network interventions for depression in young people [19]. However, this review did not investigate other mental health conditions in young people. Other systematic reviews have investigated the effectiveness of health-related online peer support networks [11], and the impact of Internet support groups on depressive symptoms [18]. These reviews, however, did not focus on young people and mental health problems in general.

Therefore, it is unclear whether online peer-to-peer support is beneficial for young people and their mental health. The aim of the current review is to systematically evaluate the evidence regarding the effectiveness of online peer-to-peer support for young people with mental health problems.

## Methods

### Databases

PubMed, PsycInfo, and Cochrane databases were searched using keywords, phrases, and Medical Subject Headings (MeSH) terms in June 2014 (see Multimedia Appendix 1).

### Search Methodology

The search strategy covered the following concepts: (1) technology, online communities, and methods of peer-to-peer interaction; (2) young people; and (3) mental health. Search terms regarding concept (1) were based on those used by Eysenbach et al [11], and Griffiths et al [18]. Search terms regarding concept (2) (young people) were developed by the researchers. Search terms regarding concept (3) (mental health) were based on the International Classification of Diseases (ICD-10) list of mental disorders and the National Health and Medical Research Council (NHMRC) keywords for mental health research [20]. The current systematic review follows the Preferred Reporting Items for Systematic Reviews and Meta-Analyses (PRISMA) statement [21]. A PRISMA checklist is available in Multimedia Appendix 2.

### Study Identification

#### Overview

**Figure 1:**
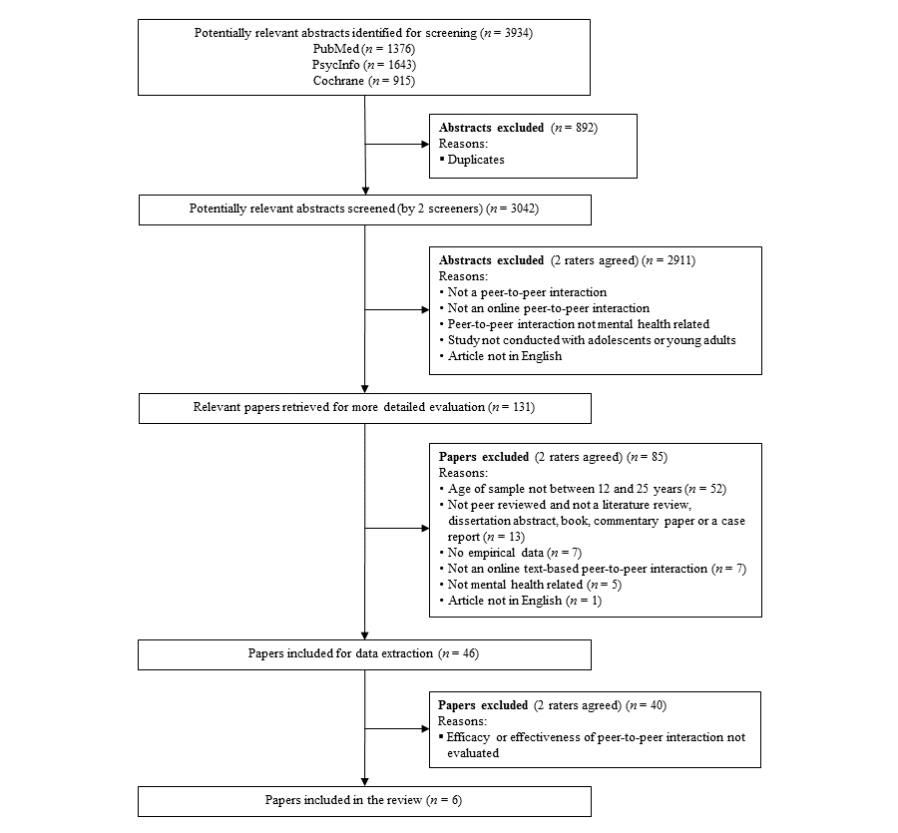
Study identification flowchart.

Figure 1 presents the flowchart for the selection of included studies. A process involving three screening stages was applied to select relevant studies for the present review. In total, the database searches yielded 3934 abstracts, of which 892 duplicates were removed.

#### Stage 1 Screening

For the first stage, in order to eliminate clearly irrelevant abstracts, two independent raters (KA and BC or RR) screened the remaining 3042 abstracts for relevant studies according to the following inclusion criteria (Textbox 1). This first screening stage yielded a total of 131 relevant papers.

#### Stage 2 Screening

In the second stage, the inclusion criteria were refined (Textbox 2) and the remaining 131 papers were screened according to these criteria by two independent raters (KA and LF or AG).

**Inclusion criteria for the fist stage.**

1. The study discussed or investigated a peer-to-peer interaction.
2. The study discussed or investigated at least one of the following: online/electronic support groups, online/electronic social or peer support, online computer-based communication or interaction, collaborative virtual environments or interventions.
3. The peer-to-peer interaction focused on mental health or psychology related conditions (eg, mental illness, smoking, etc).
4. The study sample was composed of adolescents (12-17) or young adults (18-25).
5. The article was written in English.

**Inclusion criteria for the second stage.**

1. Age: the mean age or the age range of the sample was between 12 to 25 years. If sample age was not specified in the paper, studies that related to students were included.
2. Peer-to-peer interaction: the peer-to-peer interaction was online and text-based. Studies that involved interactions in virtual reality environments that were not also text-based did not satisfy this criterion.
3. Peer-to-peer focus: the peer-to-peer interaction discussed or investigated mental health or psychology related conditions.
4. The study reported empirical data.
5. The study was peer-reviewed and not a literature review, dissertation abstract, book, commentary paper or a case report.
6. The article was in English.

#### Stage 3 Screening

A total of 46 papers were retained for the third screening stage and were included if they met the following criterion, rated by KA and LF: The study evaluated the effectiveness of peer-to-peer interaction, either as a stand-alone intervention or as a component of an intervention. Component intervention studies (eg, online cognitive behavioral therapy plus a support group) where the effectiveness of the peer support component of the intervention could not be isolated were excluded.

In addition, previous reviews, key journals, and reference lists of key papers were hand searched. However, no eligible studies were identified using this method. Finally, six papers were included for coding by two coders (KA and LF). At all stages of abstract screening, studies that raters mutually agreed on were retained and any disagreement was resolved by discussion.

### Coding of Included Papers

The six papers evaluating the effectiveness of peer-to-peer interaction were coded independently by two raters with a preformulated coding sheet. Included studies were coded for (1) participant characteristics, (2) intervention design, (3) peer-to-peer support interaction, and (4) study design characteristics. Data coding of *participant characteristics* comprised the following: participant type, symptom level of recruited participants, sample size, age, and sex. Data coding regarding the *intervention design* and *peer-to-peer support interaction* included the following: intervention description, the format of the peer-to-peer interaction (Internet support groups/discussion groups/bulletin boards/forums, chatrooms, virtual reality), peer support type (public, research, other), and whether and by whom (consumers/health professionals/unknown) it was moderated. Finally, coding of *study design characteristics* included study design (randomized controlled trial [RCT]/randomized trial/pre-post), whether or not intention-to-treat (ITT) analysis was employed, dropout (n, %), the primary outcome measure for the study, measurement time points, whether or not the intervention yielded a statistically significant positive outcome and where possible Hedge’s *g* effect sizes for differences between the intervention and the control group post-intervention.

In order to evaluate study quality, the risk of bias criteria proposed by the Cochrane Effective Practice and Organisation of Care Group (EPOC) was used [22]. These criteria are designed to assess potential sources of bias for studies involving a control group. The EPOC criteria assess the following nine study characteristics: random allocation sequence and allocation concealment, differences in baseline outcome measurements and characteristics, treatment of missing outcome data, researcher knowledge of allocated interventions, contamination between conditions, selective outcome reporting, and any other risk of bias.

### Data Analysis

Given the small number and the heterogeneous nature of the studies, a quantitative meta-analysis was not undertaken. Study results pertaining to the primary outcome measure(s) of each study were reported. For the randomized controlled trials and the randomized trial, between group results were reported, including the group by time interaction. For pre-post studies, within group results were reported. Where studies reported mean scores and standard deviations for the primary outcome measures, between group Hedge’s *g* corrections for small sample size [23] and confidence intervals were calculated for the randomized controlled trials and the randomized trial. One of the randomized controlled trials and both of the pre-post studies contained insufficient data to calculate effect sizes.

##  Results

### Study Characteristics

Detailed characteristics of the included studies (n=6) are provided in Multimedia Appendix 3. Half of the studies were RCTs [24-26], two studies used a pre-post design [27,28] and one study was a randomized trial without a control group [29]. All three RCTs employed a no-intervention control group. Sample sizes ranged from 26 to 283 (median=95) across all studies. The studies were categorized according to the mental health topic of the online peer support network. The conditions targeted were depression and anxiety [26,27], general psychological problems [29], eating disorders [24], and substance use (tobacco) [25,28]. The symptom level at baseline included low to moderate levels of psychological distress, depressive symptoms, psychological problems, and regular smoking.

### Origin

Half of the studies were conducted in the United States (n=3), and the remaining studies were from Australia, England, and Ireland.

### Interventions

The majority of studies employed Internet support groups, bulletin boards, or forums (n=4), and the remaining studies focused on virtual reality chat (n=2). Most of the studies investigated peer-to-peer support platforms that were developed for research purposes and not available to the public. Five studies reported that support groups were moderated by either health professionals or consumers. The intervention length ranged from 3 to 10 weeks with a mean of 6.8 (SD 2.3) and the length of the longest follow-up ranged from 1-12 months post-intervention.

### Participants

Most of the samples consisted of university students (n=4), and the remaining studies included rural teens or adolescent smokers. The range of mean age of participants in the sample fell between 15 to 21 years. Most of the studies targeted young adults (n=4) aged 18 to 25. In half of the studies (n=3), either all or a majority of the participants were female; one study contained equal numbers of males and females, one study contained predominantly males, and one study did not report participant gender. In most studies, participants were recruited at universities or schools (n=5).

### Outcome Measures

The two studies targeting depression and anxiety used the Depression Anxiety Stress Scale (DASS) or the Center for Epidemiologic Studies Depression Scale (CES-D) as their primary outcome measure. The single study targeting psychological stress used the Clinical Outcomes in Routine Evaluation-Outcome Measure (CORE-OM). One study targeting eating disorders used the Eating Disorder Inventory (EDI), and the two remaining studies targeting substance use problems (tobacco) used past-week abstinence rates.

### Study Quality

Of the six included studies, three studies reported completer analyses; two used an ITT design, and one study reported completer analyses for all outcomes and ITT analyses for some outcomes. Among the studies reporting ITT analyses, one study reported data from a full sample (no dropouts), one study estimated missing data using a generalized estimating equations approach, and one study used baseline scores to estimate missing data. Among all samples dropout ranged from to 0% to 86%.

Table 1 displays the ratings for the EPOC quality criteria for studies involving a control group [24-26,29]. None of the studies applied or indicated adequate randomization methods. Half of the studies indicated significant differences in the outcome measures across study groups at baseline. More than half of the studies reported that baseline characteristics of the study and control providers were similar. More than half of the studies reported that they used measures to adequately address incomplete data. None of the studies reported that the knowledge of the allocated interventions was adequately prevented during the study. In contrast, almost all studies reported that the study was adequately protected against contamination, that the study was free from selective outcome reporting and that the study was free from other risks of bias.

**Table 1:** Details of studies meeting quality rating criteria.

Study	Ellis 2011 [26]	Freeman 2008 [29]	Low 2006 [24]	Woodruff 2007 [25]
Was the allocation sequence adequately generated?				
Was the allocation adequately concealed?				
Were baseline outcome measurements similar?	✔	✔		
Were baseline characteristics similar?	✔	✔	✔	
Were incomplete outcome data adequately addressed?	✔		✔	✔
Was knowledge of the allocated interventions adequately prevented during the study?				
Was the study adequately protected against contamination?	✔	✔	✔	✔
Was the study free from selective outcome reporting?	✔	✔	✔	✔
Was the study free from other risks of bias?	✔	✔		✔

### Intervention Efficacy

#### Depression and Anxiety Symptoms

Both the RCT and the pre-post study targeting depression and anxiety symptoms examined moderated forums [26,27]. The RCT demonstrated that an online peer support forum was effective compared with the control condition at post-intervention in reducing anxiety (*g*=-.91), but not depression (*g*=-.63) [26]. In the pre-post study, a reduction in depressive symptoms between pre and post-intervention was found among users of the forum [27]. However, this reduction was not statistically significant.

#### Psychological Problems

The single study targeting psychological problems was a randomized trial and compared an electronic bulletin board plus online information with online information alone [29]. The moderation status of the bulletin board was unknown. A significant reduction in depressive symptoms was observed in both intervention groups from pre- to post-intervention. There was no evidence for an additional effect of the electronic support group post-intervention in reducing depressive symptoms (*g*=-.22).

#### Eating Disorders

The single RCT targeting eating disorders compared a no intervention control condition with three conditions, comprising the Student Bodies program plus either (1) moderated peer support, (2) unmoderated peer support, or (3) no peer support [24]. No significant differences were found between the three groups, post-intervention and at follow-up, indicating that no additional effect of peer-to-peer support to the Student Bodies program was found. Between group effect sizes for Bulimia, Body Dissatisfaction and Drive for Thinness subscales ranged from -1.05 to 0.98.

#### Smoking

The RCT and the pre-post study targeting tobacco use, both examined virtual world chat rooms [25,28]. While in both studies participants were smokers at baseline, the RCT reported significantly higher abstinence rates in the intervention group compared to the control group post intervention [25]. The pre-post study showed an increase in smoking abstinence (11%) in the past week from pre- to post-intervention, although this change was not significant [28].

##  Discussion

### Principal Findings

This systematic review identified six studies (3 RCTs, 2 pre-post, 1 randomized trial) examining the effectiveness of online peer-to-peer support for young people with mental health problems. The studies targeted a range of mental health issues, including depression and anxiety, general psychological problems, eating disorders and substance use (tobacco). Overall, two of the 4 RCTs/randomized trials yielded a positive effect for the peer-support group relative to the comparison group at post-intervention: the RCT targeting anxiety and the RCT targeting tobacco [25,26]. There was no evidence that peer-to-peer support was effective for eating disorder or depressive symptoms [24,26]. However, the study targeting depressive symptoms might have been underpowered given the magnitude of the effect size. Of the two trials that yielded positive effects, one used a moderated discussion group [26] and the other utilized virtual reality chat [25]. In general, studies were of low quality, scoring 4.6 out of 9 on average.

Thus, the current review found some evidence for the efficacy of peer-to-peer support alone or as an adjunct to other treatment programs for mental health problems in young people. These findings are similar to a previous systematic review targeting online and social networking interventions in young people for depression [19], which reported positive outcomes for some of the social networking studies. The results are also consistent with findings from two studies of ISGs that have targeted adults with depression [30,31]. It is encouraging in the present review that two of the interventions tested in randomized controlled trials were shown to be effective in comparison to the control groups. Although possibly limited in their generalizability due to limited sample sizes, high numbers of female participants, large variations in dropout rates and specific mental health problems (anxiety and tobacco) these findings are of interest, particularly given the extensive use of peer support in the field of mental health [16].

The majority of discussion groups were moderated by health professionals, researchers or consumers. However, there was limited information on moderators, their level of skills, and their engagement with the discussion group. In addition, although the type of moderation might impact the outcome for participants, included studies did not provide detailed information on the use of theory driven moderation or discussion of risk management. Given that moderation is a critical aspect of peer-to-peer support, future studies should include details about the type of moderation and moderators. This may shed light on which level of moderation works best for participants.

The paucity of high-quality randomized controlled trials examining the effects of peer support in young people makes it difficult to draw firm conclusions about its effectiveness. These findings are similar to previous systematic reviews of online peer-to-peer support in adults. For example, one review found that all identified studies that evaluated the additional effect of peer support were pre-post studies with low-quality research designs [11]. Another review that focused on Internet support groups and depression in adults found similar results, emphasizing the need for high-quality research in this field [18]. The lack of high-quality studies is especially concerning, given young people’s extensive use of the Internet to search for mental health information and connect with others [12].

From this review it is clear that research on peer-to-peer support among young people has been dominated by studies that employed asynchronous communication. In this review only two studies investigated synchronous communication; both involved a virtual reality chat component targeting tobacco. There is a need to investigate the effectiveness of synchronous chat sessions for other mental health problems in young people.

It is also important to note that although peer support is frequently used as an adjunct to online interventions, very few studies have isolated and investigated the additional effect of online peer support. The present review addressed this specific gap in the literature. This resulted in the exclusion of 40 studies. It is disappointing that so few studies sought to identify the specific contribution of peer-to-peer support. Opinions on this approach are mixed and the suitability and practicability of peer-to-peer support as an intervention in and of itself has been the subject of debate [11,32]. Qualitative studies of online peer support contribute to understanding user characteristics, perceived benefits, potential risks, and the self-help process [32]. Yet, the question remains under which conditions and for whom these support groups are effective and how social support can be improved [11]. Future studies should use both qualitative and quantitative methods to investigate this question.

The field of online peer-to-peer support is still in its infancy and many questions remain unanswered. Despite this fact, given that many online interventions for young people include a peer support component, there is an urgent need to distinguish which parts of these interventions are effective.

### Limitations

There are several limitations to this systematic review. Studies were identified based on searches in three databases. It is possible that this search strategy failed to identify some eligible studies. To address this in part, previous reviews, key journals, and key papers were hand searched. Despite extensive search, no additional eligible studies were identified using this method. A further level of bias might be due to the criterion that only English language papers were included in the present review. Finally, the review may be subject to publication bias if authors failed to publish some studies with null findings.

### Implications for Future Research

High-quality research on online peer-to-peer support for young people is currently lacking. Many studies examining Internet interventions for young people use peer support as an adjunct. However, there is limited evidence for the effectiveness of this addition to care. It is vital that future research explores the specific contribution of peer support to these interventions. Based on the findings published to date, it is not possible to conclude for whom and under which conditions peer support interactions work. In addition, the absence of studies involving individuals experiencing severe problems or in recovery shows it is not known if peer-to-peer support is appropriate for this group.

In summary, given that a majority of young people are using the Internet routinely, further research is needed to explore the role that peer-to-peer support might play in assisting young people with mental health problems.

## Appendix

Multimedia Appendix 1 Search terms/search history.

Multimedia Appendix 2 PRISMA checklist.

Multimedia Appendix 3 Full data from the review.

## Abbreviations

CES-D: Center for Epidemiologic Studies Depression Scale
CORE-OM: Clinical Outcomes in Routine Evaluation-Outcome Measure
DASS: Depression Anxiety Stress Scale
EDI: Eating Disorder Inventory
EPOC: Effective Practice and Organisation of Care Group
ICD-10: International Classification of Diseases
ISGs: Internet support groups
ITT: Intention-to-Treat
MeSH: Medical Subject Headings
NHMRC: National Health and Medical Research Council
PRSIMA: Preferred Reporting Items for Systematic Reviews and Meta-Analyses
RCT: randomized controlled trial

## References

[1] Patel Vikram, Flisher Alan J, Hetrick Sarah, McGorry Patrick (2007). Mental health of young people: a global public-health challenge. Lancet.

[2] Australian Institute of Health and Welfare (2011). Young Australians: their health and wellbeing.

[3] Booth Michael L, Bernard Diana, Quine Susan, Kang Melissa S, Usherwood Tim, Alperstein Garth, Bennett David L (2004). Access to health care among Australian adolescents young people's perspectives and their sociodemographic distribution. J Adolesc Health.

[4] Rickwood D, Deane FP, Wilson CJ, Ciarrochi J (2005). Young people’s help-seeking for mental health problems. Australian e-Journal for the Advancement of Mental Health.

[5] Wetterlin FM, Mar MY, Neilson EK, Werker GR, Krausz M (2014). eMental health experiences and expectations: a survey of youths' Web-based resource preferences in Canada. J Med Internet Res.

[6] Lenhart A, Purcell K, Smith A, Zickuhr K Social media & mobile internet use among teens and young adults.

[7] Bauer Stephanie, Moessner Markus (2013). Harnessing the power of technology for the treatment and prevention of eating disorders. Int J Eat Disord.

[8] Campbell AJ, Robards F Using technologies safely and effectively to promote young people's wellbeing: a better practice guide for services.

[9] Griffiths Km, Christensen H (2007). Internet-based mental health programs: A powerful tool in the rural medical kit. Aust J Rural Health.

[10] King R, Bickman L, Shochet I, McDermott B, Bor B (2010). Use of the internet for provision of better counselling and psychotherapy services to young people, their families and carers. Psychotherapy in Australia.

[11] Eysenbach Gunther, Powell John, Englesakis Marina, Rizo Carlos, Stern Anita (2004). Health related virtual communities and electronic support groups: systematic review of the effects of online peer to peer interactions. BMJ.

[12] Burns Jane M, Davenport Tracey A, Durkin Lauren A, Luscombe Georgina M, Hickie Ian B (2010). The internet as a setting for mental health service utilisation by young people. Med J Aust.

[13] Tanis Martin (2008). Health-related on-line forums: what's the big attraction?. J Health Commun.

[14] Wright Kevin B, Bell Sally B, Wright Kevin B, Bell Sally B (2003). Health-related Support Groups on the Internet: Linking Empirical Findings to Social Support and Computer-mediated Communication Theory. J Health Psychol.

[15] Batenburg Anika, Das Enny (2014). Emotional approach coping and the effects of online peer-led support group participation among patients with breast cancer: a longitudinal study. J Med Internet Res.

[16] Barak A, Dolev-Cohen M (2006). Does activity level in online support groups for distressed adolescents determine emotional relief. Counselling and Psychotherapy Research.

[17] Pfeiffer Paul N, Heisler Michele, Piette John D, Valenstein Marcia, Rogers Mary A M (2011). Efficacy of peer support interventions for depression: a meta-analysis. Gen Hosp Psychiatry.

[18] Griffiths Kathleen M, Calear Alison L, Banfield Michelle (2009). Systematic review on Internet Support Groups (ISGs) and depression (1): Do ISGs reduce depressive symptoms?. J Med Internet Res.

[19] Rice Simon M, Goodall Joanne, Hetrick Sarah E, Parker Alexandra G, Gilbertson Tamsyn, Amminger G Paul, Davey Christopher G, McGorry Patrick D, Gleeson John, Alvarez-Jimenez Mario (2014). Online and social networking interventions for the treatment of depression in young people: a systematic review. J Med Internet Res.

[20] National Health and Research Council (NHMRC).

[21] Moher David, Liberati Alessandro, Tetzlaff Jennifer, Altman Douglas G (2009). Preferred reporting items for systematic reviews and meta-analyses: the PRISMA statement. Ann Intern Med.

[22] Effective Practice Organisation of Care (EPOC) Suggested risk of bias criteria for EPOC reviews.

[23] Borenstein M (2009). Effect sizes for continuous data. Cooper H, Hedges LV, Valentine JC, editors. The handbook of research synthesis and meta-analysis.

[24] Low Kathryn Graff, Charanasomboon Swita, Lesser Jill, Reinhalter Katie, Martin Rachel, Jones Hannah, Winzelberg Andy, Abascal Liana, Taylor C Barr (2006). Effectiveness of a computer-based interactive eating disorders prevention program at long-term follow-up. Eat Disord.

[25] Woodruff Susan I, Conway Terry L, Edwards Christine C, Elliott Sean P, Crittenden Jim (2007). Evaluation of an Internet virtual world chat room for adolescent smoking cessation. Addict Behav.

[26] Ellis LA, Campbell AJ, Sethi S, O'Dea BM (2011). Comparative randomized trial of an online cognitive-behavioral therapy program and an online support group for depression and anxiety. J Cyber Ther Rehabil.

[27] Horgan Aine, McCarthy Geraldine, Sweeney John (2013). An evaluation of an online peer support forum for university students with depressive symptoms. Arch Psychiatr Nurs.

[28] Woodruff S I, Edwards C C, Conway T L, Elliott S P (2001). Pilot test of an Internet virtual world chat room for rural teen smokers. J Adolesc Health.

[29] Freeman E, Barker C, Pistrang N (2008). Outcome of an online mutual support group for college students with psychological problems. Cyberpsychol Behav.

[30] Houston Thomas K, Cooper Lisa A, Ford Daniel E (2002). Internet support groups for depression: a 1-year prospective cohort study. Am J Psychiatry.

[31] Griffiths Kathleen M, Mackinnon Andrew J, Crisp Dimity A, Christensen Helen, Bennett Kylie, Farrer Louise (2012). The effectiveness of an online support group for members of the community with depression: a randomised controlled trial. PLoS One.

[32] Barak A, Grohol JM, Pector E BMJ.

