# Supplementary material for: Online Peer-to-Peer Support for Young People With Mental Health Problems: A Systematic Review
Source: JMIR Ment Health. 2015 May 19;2(2):e19. doi: 10.2196/mental.4418 (PMC4607385; doi:10.2196/mental.4418)
Supplement: Multimedia Appendix 1 [file mental_v2i2e19_app1.pdf]

Multimedia Appendix 1. Search terms/search history.

Database/Notes

Terms

Results

PubMed

#1

"computer communication network"[Title/Abstract] OR "computer-network"[Title/Abstract] OR "computer-based"[Title/Abstract] OR "computer-generated environment"[Title/Abstract] OR cyber[Title/Abstract] OR cyberspace[Title/Abstract] OR "data highway"[Title/Abstract] OR electronic[Title/Abstract] OR "electronic mail"[Title/Abstract] OR email[Title/Abstract] OR "e-mail"[Title/Abstract] OR "information highway"[Title/Abstract] OR "information superhighway"[Title/Abstract] OR "information super highway"[Title/Abstract] OR internet[Title/Abstract] OR "internet-based"[Title/Abstract] OR net[Title/Abstract] OR online[Title/Abstract] OR virtual[Title/Abstract] OR web[Title/Abstract] OR "web-based"[Title/Abstract] OR webbased[Title/Abstract] OR "web page"[Title/Abstract] OR webpage[Title/Abstract] OR "web site"[Title/Abstract] OR website[Title/Abstract] OR "world wide web"[Title/Abstract] OR www[Title/Abstract]

349775

#2

Search "discussion group"[Title/Abstract] OR forum[Title/Abstract] OR "interpersonal communication"[Title/Abstract] OR "interpersonal interaction"[Title/Abstract] OR "multi-user"[Title/Abstract] OR network[Title/Abstract] OR "peer support"[Title/Abstract] OR "peer-support"[Title/Abstract] OR "peer to peer"[Title/Abstract] OR p2p[Title/Abstract] OR selfhelp[Title/Abstract] OR "self-help"[Title/Abstract] OR "social network"[Title/Abstract] OR "social support"[Title/Abstract] OR "support group"[Title/Abstract] OR "support network"[Title/Abstract] OR "support system"[Title/Abstract]

230429

#3

Search (#1 AND #2)

15979

#4

Search ("internet community"[Title/Abstract] OR "internet-based community"[Title/Abstract] OR "world wide web community"[Title/Abstract] OR "e-mail community"[Title/Abstract] OR "email community"[Title/Abstract] OR "electronic mail community"[Title/Abstract] OR "web community"[Title/Abstract] OR "web page community"[Title/Abstract] OR "webpage community"[Title/Abstract] OR "web site community"[Title/Abstract] OR "website community"[Title/Abstract] OR "web-based community"[Title/Abstract] OR "webbased community"[Title/Abstract] OR "www community"[Title/Abstract] OR

"cyber community"[Title/Abstract] OR "virtual community"[Title/Abstract] OR "online community"[Title/Abstract] OR "e-community"[Title/Abstract] OR "electronic community"[Title/Abstract])

361

#5

Search ("internet society"[Title/Abstract] OR "internet-based society"[Title/Abstract] OR "world wide web society"[Title/Abstract] OR "e-mail society"[Title/Abstract] OR "email society"[Title/Abstract] OR "electronic mail society"[Title/Abstract] OR "web society"[Title/Abstract] OR "web page society"[Title/Abstract] OR "webpage society"[Title/Abstract] OR "web site society"[Title/Abstract] OR "website society"[Title/Abstract] OR "web-based society"[Title/Abstract] OR "webbased society"[Title/Abstract] OR "www society"[Title/Abstract] OR "cyber society"[Title/Abstract] OR "virtual society"[Title/Abstract] OR "online society"[Title/Abstract] OR “e-society"[Title/Abstract] OR "electronic society"[Title/Abstract])

4

#6

Search ("internet environment"[Title/Abstract] OR "internet-based environment"[Title/Abstract] OR "world wide web environment"[Title/Abstract] OR "e-mail environment"[Title/Abstract] OR "email environment"[Title/Abstract] OR "electronic mail environment"[Title/Abstract] OR "web environment"[Title/Abstract] OR "web page environment"[Title/Abstract] OR "webpage environment"[Title/Abstract] OR "web site environment"[Title/Abstract] OR "website environment"[Title/Abstract]

OR "web-based environment"[Title/Abstract] OR "webbased environment"[Title/Abstract] OR "www environment"[Title/Abstract] OR "cyber environment"[Title/Abstract] OR "virtual environment"[Title/Abstract] OR "online environment"[Title/Abstract] OR "e-environment"[Title/Abstract] OR "electronic environment"[Title/Abstract])

1716

#7

Search ("bulletin board system"[Title/Abstract] OR "chat group"[Title/Abstract] OR chatgroup[Title/Abstract] OR "chat room"[Title/Abstract] OR chatroom[Title/Abstract] OR "chat technology"[Title/Abstract] OR "discussion list"[Title/Abstract] OR "ebulletin board"[Title/Abstract] OR "e-bulletin board"[Title/Abstract] OR "electronic bulletin board"[Title/Abstract] OR "instant messaging"[Title/Abstract] OR "internet relay"[Title/Abstract] OR irc[Title/Abstract] OR "list serv"[Title/Abstract] OR listserv[Title/Abstract] OR "mail box"[Title/Abstract] OR mailbox[Title/Abstract] OR "mailing list"[Title/Abstract] OR "message board"[Title/Abstract] OR messageboard[Title/Abstract] OR "news group"[Title/Abstract] OR newsgroup[Title/Abstract] OR usenet[Title/Abstract])

1526

#8

Search ("computer-based group"[Title/Abstract] OR "computer-based support"[Title/Abstract] OR "computer-based interaction"[Title/Abstract] OR "computer-based intervention"[Title/Abstract] OR "cyber group"[Title/Abstract] OR "cyber support"[Title/Abstract] OR "cyber interaction"[Title/Abstract] OR "cyber intervention"[Title/Abstract] OR "electronic support"[Title/Abstract] OR "electronic group"[Title/Abstract] OR "electronic interaction"[Title/Abstract] OR "electronic intervention"[Title/Abstract] OR "e-support"[Title/Abstract] OR "e-group"[Title/Abstract] OR "e-interaction"[Title/Abstract]

OR "e-intervention"[Title/Abstract] OR "internet group"[Title/Abstract] OR "internet support"[Title/Abstract] OR "internet-based group"[Title/Abstract] OR "internet-based support"[Title/Abstract] OR "online group"[Title/Abstract] OR "online support"[Title/Abstract] OR "online network"[Title/Abstract] OR "virtual support"[Title/Abstract] OR "virtual group"[Title/Abstract] OR "virtual interaction"[Title/Abstract] OR "virtual intervention"[Title/Abstract] OR "web group"[Title/Abstract] OR "web support"[Title/Abstract] OR "web interaction"[Title/Abstract] OR "web intervention"[Title/Abstract] OR "web-based group"[Title/Abstract] OR "web-based support"[Title/Abstract] OR "web-based interaction"[Title/Abstract] OR "web-based intervention"[Title/Abstract] OR "web-based program"[Title/Abstract] OR "online interaction"[Title/Abstract] OR "online intervention"[Title/Abstract])

3388

#9

Search (#3 OR #4 OR #5 OR #6 OR #7 OR #8)

22167

#10

Search (youth*[Title/Abstract] OR child*[Title/Abstract] OR adolescen*[Title/Abstract] OR teen*[Title/Abstract] OR young*[Title/Abstract] OR juvenile[Title/Abstract] OR student[Title/Abstract] OR school[Title/Abstract] OR college[Title/Abstract] OR university[Title/Abstract])

1818986

#11

Youth Terms

Search (#9 AND #10)

3155

#12

Youth Limits

Search #3 OR #4 OR #5 OR #6 OR #7 OR #8 Filters: Child: 6-12 years; Adolescent: 13-18 years; Young Adult: 19-24 years

2251

#13

Combined

Search (#11 OR #12)

4318

#14

Mental Health

Search ("mental health" OR "mental disorder*" OR psychiatr* OR "mental disease" OR "neuropsychiatric disorder*" OR psychopathology OR "domestic violence" OR fear OR "addiction" OR "alcohol dependence" OR "substance use" OR

"substance abuse" OR intoxication OR "harmful use" OR withdrawal OR alcohol OR opioid* OR cannabinoids OR cannabis OR

sedatives OR hypnotics OR cocaine OR stimulants OR caffeine OR hallucinogens OR tobacco OR "volatile solvents" OR "drug use" OR "drug abuse" OR "drug dependen*" OR "drug relapse" OR "drugs of dependence" OR "opioid dependen*" OR "smoking cessation" OR "smoking intervention" OR "tobacco control" OR "tobacco dependen*" OR "tobacco use" OR amphetamine OR "crystal meth*" OR GHB OR heroin OR ice OR marijuana OR MDMA OR methamphetamine* OR polysubstance OR phencyclidine OR Schizophrenia OR schizotypal OR schizophreniform OR schizoaffective OR schizoid OR paranoi* OR delusion* OR psychosis OR psychotic OR paraphrenia OR "affective disorder" OR "affective symptom*" OR "mood disorder*" OR depress* OR dysthymi* OR MDD OR CBT OR "cognitive behavior*" OR "cognitive behaviour*" OR IPT OR PST OR DBT OR psychotherapy OR stress OR "stress disorder" OR neurosis OR neurotic OR manic OR mania OR hypomania* OR cyclothymi* OR bipolar OR anxiety OR panic OR agoraphobi* OR "social phobia" OR "generalized anxiety disorder" OR GAD OR "obsessive compulsive" OR OCD OR "adjustment disorder" OR "separation anxiety" OR "post-traumatic stress" OR PTSD OR phobi* OR neurasthenia OR somatoform OR somatization OR "pain disorder*" OR hypochondria* OR hysteria OR dissociat* OR "depersonalization disorder" OR "personality disorder*" OR "borderline personality" OR BPD OR "antisocial personality" OR APD OR anankastik OR "dissocial personality" OR "dyssocial personality" OR "dependent personality" OR "anxious personality" OR "avoidant personality" OR histrionic OR narcissis* OR "passive-aggressive" OR "personality change" OR "eating disorder*" OR anorexia OR bulimia OR "body dysmorph*" OR "conversion disorder" OR "attention deficit" OR ADHD OR hyperkinetic OR hyperactive* OR impulsive* OR instability OR "Mood shifts" OR PDD-NOS OR autis* OR asperger* OR bully* OR "conduct disorder" OR "oppositional defiant" OR "emotional disorder" OR

"separation disorder" OR tic OR tics OR tourette OR pica OR "stereotyped movement disorder*" OR "mild depressive disorder" OR internaliz* OR externaliz* OR internalis* OR externalis* OR "child abuse" OR "childhood disintegrative disorder" OR "pervasive developmental" OR compulsive OR delinquent OR aggress* OR "learning disorder*" OR "developmental disabilit*" OR "repetitive behaviours deficit" OR enuresis OR encopresis OR "mental retardation" OR "intellectual disability*" OR "behaviour* problem*" OR apprehension OR stuttering OR stammering OR cluttering OR "selective mutism" OR "attachment disorder" OR "sleep disorder*" OR insomnia OR hypersomnia OR dyssomnia OR parasomnia OR "sleep terror" OR sleepwalking OR gambl* OR pyromania OR kleptomania OR "sexual dysfunction" OR "tension headache" OR

"sexual disorder" OR dyspareunia OR exhibitionism OR "orgasmic disorder" OR fetishism OR frotteurism OR "gender identity disorder" OR "impulse-control disorder" OR "intermittent explosive disorder" OR "erectile disorder" OR "nightmare" OR paraphilia OR pedophilia OR paedophilia OR "premature ejaculation" OR pyromania OR "sexual aversion disorder" OR "sexual masochism" OR "sexual sadism" OR sadomasochism OR "sexual maturation disorder" OR "transvestic fetishism" OR "egodystonic sexual orientation" OR "sexual relationship disorder" OR "sexual arousal disorder" OR dyspareunia OR trichotillomania OR vaginismus OR voyeurism OR suicid* OR self injur* OR "self harm" OR violen* OR offend* OR "Factitious disorder" OR munchausen OR "ganser syndrome")

3743776

#15

Combined AND Mental Health

Search (#13 AND #14)

1376

PsycInfo

#1

(computer communication network or computer-network or computer-based or computer-generated environment or cyber or cyberspace or data highway or electronic or electronic mail or email or e-mail or information highway or information super highway or information superhighway or internet or internet-based or net or online or virtual or web or web-based or webbased or web page or webpage or web site or website or world wide web or www).ab,ti.

97676

#2

(discussion group or forum or interpersonal communication or interpersonal interaction or multi-user or network or peer-support or peer support or peer to peer or p2p or selfhelp or self-help or social network or social support or support network or support group or support system).ab,ti.

98510

#3

1 and 2

7647

#4

(internet community or internet-based community or world wide web community or e-mail community or email community or electronic mail community or web community or web-based community or webbased community or web page community or webpage community or website community or web site community or www community or cyber community or virtual community or online community or e-community or electronic community).ab,ti.

821

#5

(internet society or internet-based society or world wide web society or e-mail society or email society or electronic mail society or web society or web page society or webpage society or web site society or website society or web-based society or webbased society or www society or cyber society or virtual society or online society or e-society or electronic society).ab,ti.

36

#6

(internet environment or internet-based environment or world wide web environment or e-mail environment or email environment or electronic mail environment or web environment or web page environment or webpage environment or web site environment or website environment or web-based environment or webbased environment or www environment or cyber environment or virtual environment or online environment or e-environment or electronic environment).ab,ti.

2121

#7

(bulletin board system or chat group or chatgroup or chat room or chatroom or chat technology or discussion list or ebulletin board or e-bulletin board or electronic bulletin board or instant messaging or internet relay or irc or list serv or listserv or mail box or mailbox or mailing list or message board or messageboard or news group or newsgroup or usenet).ab,ti.

1420

#8

(computer-based group or computer-based support or computer-based interaction or computer-based intervention or cyber group or cyber support or cyber interaction or cyber intervention or electronic support or electronic group or electronic interaction or electronic intervention or e-support or e-group or e-interaction or e-intervention or internet group or internet support or internet-based group or internet-based support or online group or online support or online interaction or online intervention or online network or virtual support or virtual group or virtual interaction or virtual intervention or web group

or web support or web interaction or web intervention or web-based group or web-based support or web-based interaction or web-based intervention or web-based program).ab,ti.

1806

#9

3 or 4 or 5 or 6 or 7 or 8

12696

#10

Youth limits

limit 64 to (180 school age <age 6 to 12 yrs> or 200 adolescence <age 13 to 17 yrs> or 320 young adulthood <age 18 to 29 yrs>)

2899

#11

(youth$ or child$ or adolescen$ or teen$ or young$ or juvenile or student or school or college or university).ab,ti.

1043654

#12

Youth terms

9 and 11

3809

#13

Combined

10 or 13

5271

#14

Mental Health

("mental health" OR "mental disorder*" OR psychiatr* OR "mental disease" OR "neuropsychiatric disorder*" OR psychopathology OR "domestic violence" OR fear OR "addiction" OR "alcohol dependence" OR "substance use" OR "substance abuse" OR intoxication OR "harmful use" OR withdrawal OR alcohol OR opioid* OR cannabinoids OR cannabis OR

sedatives OR hypnotics OR cocaine OR stimulants OR caffeine OR hallucinogens OR tobacco OR "volatile solvents" OR "drug use" OR "drug abuse" OR "drug dependen*" OR "drug relapse" OR "drugs of dependence" OR "opioid dependen*" OR "smoking cessation" OR "smoking intervention" OR "tobacco control" OR "tobacco dependen*" OR "tobacco use" OR amphetamine OR "crystal meth*" OR GHB OR heroin OR ice OR marijuana OR MDMA OR methamphetamine* OR polysubstance OR phencyclidine OR Schizophrenia OR schizotypal OR schizophreniform OR schizoaffective OR schizoid OR paranoi* OR delusion* OR psychosis OR psychotic OR paraphrenia OR "affective disorder" OR "affective symptom*" OR

"mood disorder*" OR depress* OR dysthymi* OR MDD OR CBT OR "cognitive behavior*" OR "cognitive behaviour*" OR IPT OR PST OR DBT OR psychotherapy OR stress OR "stress disorder" OR neurosis OR neurotic OR manic OR mania OR hypomania* OR cyclothymi* OR bipolar OR anxiety OR panic OR agoraphobi* OR "social phobia" OR "generalized anxiety disorder" OR GAD OR "obsessive compulsive" OR OCD OR "adjustment disorder" OR "separation anxiety" OR "post-traumatic stress" OR PTSD OR phobi* OR neurasthenia OR somatoform OR somatization OR "pain disorder*" OR hypochondria* OR hysteria OR dissociat* OR "depersonalization disorder" OR "personality disorder*" OR "borderline personality" OR BPD OR "antisocial personality" OR APD OR anankastik OR "dissocial personality" OR "dyssocial personality" OR "dependent personality" OR "anxious personality" OR "avoidant personality" OR histrionic OR narcissis* OR "passive-aggressive" OR "personality change" OR "eating disorder*" OR anorexia OR bulimia OR "body dysmorph*" OR "conversion disorder" OR "attention deficit" OR ADHD OR hyperkinetic OR hyperactive* OR impulsive* OR instability OR "Mood shifts" OR PDD-NOS OR autis* OR asperger* OR bully* OR "conduct disorder" OR "oppositional defiant" OR "emotional disorder" OR

"separation disorder" OR tic OR tics OR tourette OR pica OR "stereotyped movement disorder*" OR "mild depressive disorder" OR internaliz* OR externaliz* OR internalis* OR externalis* OR "child abuse" OR "childhood disintegrative disorder" OR "pervasive developmental" OR compulsive OR delinquent OR aggress* OR "learning disorder*" OR "developmental disabilit*" OR "repetitive behaviours deficit" OR enuresis OR encopresis OR "mental retardation" OR "intellectual disability*" OR "behaviour* problem*" OR apprehension OR stuttering OR stammering OR cluttering OR "selective mutism" OR "attachment disorder" OR "sleep disorder*" OR insomnia OR hypersomnia OR dyssomnia OR parasomnia OR "sleep terror" OR sleepwalking OR gambl* OR pyromania OR kleptomania OR "sexual dysfunction" OR "tension headache" OR

"sexual disorder" OR dyspareunia OR exhibitionism OR "orgasmic disorder" OR fetishism OR frotteurism OR "gender identity disorder" OR "impulse-control disorder" OR "intermittent explosive disorder" OR "erectile disorder" OR "nightmare" OR paraphilia OR pedophilia OR paedophilia OR "premature ejaculation" OR pyromania OR "sexual aversion disorder" OR "sexual masochism" OR "sexual sadism" OR sadomasochism OR "sexual maturation disorder" OR "transvestic fetishism" OR "egodystonic sexual orientation" OR "sexual relationship disorder" OR "sexual arousal disorder" OR dyspareunia OR trichotillomania OR vaginismus OR voyeurism OR suicid* OR self injur* OR "self harm" OR violen* OR offend* OR "Factitious disorder" OR munchausen OR "ganser syndrome")

1347533

#15

Combined AND Mental Health

13 and 14

1643

Cochrane

#1

"computer communication network" or "computer-network" or "computer-based" or "computer-generated environment" or cyber or cyberspace or "data highway" or electronic or "electronic mail" or email or "e-mail" or "information highway" or "information superhighway" or "information super highway" or internet or "internet-based" or net or online or virtual or

web or "web-based" or webbased or "web page" or webpage or "web site" or website or "world wide web" or www

***Search: in title abstract keywords* in Cochrane Reviews (Reviews only) and Trials (Word variations have been searched)**

15293

#2

"discussion group" or forum or "interpersonal communication" or "interpersonal interaction" or

"multi-user" or network or "peer support" or "peer-support" or "peer to peer" or p2p or selfhelp or "self-help" or "social network" or "social support" or "support group" or "support network" or "support system"

***Search: in title abstract keywords* in Cochrane Reviews (Reviews only) and Trials (Word variations have been searched)**

8693

#3

#1 AND #2

1198

#4

"internet community" OR "internet-based community" OR "world wide web community" OR

"e-mail community" OR "email community" OR "electronic mail community" OR "web community" OR "web page community" OR "webpage community" OR "web site community" OR "website community" OR "web-based community" OR "webbased community" OR "www community" OR "cyber community" OR "virtual community" OR "online community" OR "e-community" OR "electronic community"

***Search: in title abstract keywords* in Cochrane Reviews (Reviews only) and Trials (Word variations have been searched)**

24

#5

"internet society" OR "internet-based society" OR "world wide web society" OR "e-mail society" OR "email society" OR "electronic mail society" OR "web society" OR "web page society" OR "webpage society" OR "web site society" OR "website society" OR "web-based society" OR "webbased society" OR "www society" OR "cyber society" OR "virtual society" OR "online society" OR “e-society" OR "electronic society"

***Search: in title abstract keywords* in Cochrane Reviews (Reviews only) and Trials (Word variations have been searched)**

0

#6

"internet environment" OR "internet-based environment" OR "world wide web environment" OR

"e-mail environment" OR "email environment" OR "electronic mail environment" OR "web environment" OR "web page environment" OR "webpage environment" OR "web site environment" OR "website environment" OR "web-based environment" OR "webbased environment" OR "www environment" OR "cyber environment" OR "virtual environment" OR "online environment" OR "e-environment" OR "electronic environment"

***Search: in title abstract keywords* in Cochrane Reviews (Reviews only) and Trials (Word variations have been searched)**

140

#7

"bulletin board system" OR "chat group" OR chatgroup OR "chat room" OR chatroom OR "chat technology" OR "discussion list" OR "ebulletin board" OR "e-bulletin board" OR "electronic bulletin board" OR "instant messaging" OR "internet relay" OR irc OR "list serv" OR listserv OR "mail box" OR mailbox OR "mailing list" OR "message board" OR messageboard OR "news group" OR newsgroup OR usenet

***Search: in title abstract keywords* in Cochrane Reviews (Reviews only) and Trials (Word variations have been searched)**

82

#8

"computer-based group" OR "computer-based support" OR "computer-based interaction" OR

"computer-based intervention" OR "cyber group" OR "cyber support" OR "cyber interaction" OR

"cyber intervention" OR "electronic support" OR "electronic group" OR "electronic interaction" OR "electronic intervention" OR "e-support" OR "e-group" OR "e-interaction" OR "e-intervention" OR "internet group" OR "internet support" OR "internet-based group" OR "internet-based support" OR "online group" OR "online support" OR "online network" OR "virtual support" OR "virtual group" OR "virtual interaction" OR "virtual intervention" OR "web group" OR "web support" OR "web interaction" OR "web intervention" OR "web-based group" OR "web-based support" OR "web-based interaction" OR "web-based intervention" OR "web-based program" OR "online interaction" OR "online intervention"

***Search: in title abstract keywords* in Cochrane Reviews (Reviews only) and Trials (Word variations have been searched)**

1033

#9

#3 OR #4 OR #5 OR #6 OR #7 OR #8

2309

#10

"mental health" OR "mental disorder*" OR psychiatr* OR "mental disease" OR "neuropsychiatric disorder*" OR psychopathology OR "domestic violence" OR fear OR "addiction" OR "alcohol dependence" OR "substance use" OR "substance abuse" OR intoxication OR "harmful use" OR withdrawal OR alcohol OR opioid* OR cannabinoids OR cannabis OR

sedatives OR hypnotics OR cocaine OR stimulants OR caffeine OR hallucinogens OR tobacco OR "volatile solvents" OR "drug use" OR "drug abuse" OR "drug dependen*" OR "drug relapse" OR "drugs of dependence" OR "opioid dependen*" OR "smoking cessation" OR "smoking intervention" OR "tobacco control" OR "tobacco dependen*" OR "tobacco use" OR amphetamine OR "crystal meth*" OR GHB OR heroin OR ice OR marijuana OR MDMA OR methamphetamine* OR polysubstance OR phencyclidine OR Schizophrenia OR schizotypal OR schizophreniform OR schizoaffective OR schizoid OR paranoi* OR delusion* OR psychosis OR psychotic OR paraphrenia OR "affective disorder" OR "affective symptom*" OR "mood disorder*" OR depress* OR dysthymi* OR MDD OR CBT OR "cognitive behavior*" OR "cognitive behaviour*" OR IPT OR PST OR DBT OR psychotherapy OR stress OR "stress disorder" OR neurosis OR neurotic OR manic OR mania OR hypomania* OR cyclothymi* OR bipolar OR anxiety OR panic OR agoraphobi* OR "social phobia" OR "generalized anxiety disorder" OR GAD OR "obsessive compulsive" OR OCD OR "adjustment disorder" OR "separation anxiety" OR "post-traumatic stress" OR PTSD OR phobi* OR neurasthenia OR somatoform OR somatization OR "pain disorder*" OR hypochondria* OR hysteria OR dissociat* OR "depersonalization disorder" OR "personality disorder*" OR "borderline personality" OR BPD OR "antisocial personality" OR APD OR anankastik OR "dissocial personality" OR "dyssocial personality" OR "dependent personality" OR "anxious personality" OR "avoidant personality" OR histrionic OR narcissis* OR

"passive-aggressive" OR "personality change" OR "eating disorder*" OR anorexia OR bulimia OR "body dysmorph*" OR "conversion disorder" OR "attention deficit" OR ADHD OR hyperkinetic OR hyperactive* OR impulsive* OR instability OR "Mood shifts" OR PDD-NOS OR autis* OR asperger* OR bully* OR "conduct disorder" OR "oppositional defiant" OR "emotional disorder" OR

"separation disorder" OR tic OR tics OR tourette OR pica OR "stereotyped movement disorder*" OR "mild depressive disorder" OR internaliz* OR externaliz* OR internalis* OR externalis* OR "child abuse" OR "childhood disintegrative disorder" OR "pervasive developmental" OR compulsive OR delinquent OR aggress* OR "learning disorder*" OR "developmental disabilit*" OR "repetitive behaviours deficit" OR enuresis OR encopresis OR "mental retardation" OR "intellectual disability*" OR "behaviour* problem*" OR apprehension OR stuttering OR stammering OR cluttering OR "selective mutism" OR "attachment disorder" OR "sleep disorder*" OR insomnia OR hypersomnia OR dyssomnia OR parasomnia OR "sleep terror" OR sleepwalking OR gambl* OR pyromania OR kleptomania OR "sexual dysfunction" OR "tension headache" OR

"sexual disorder" OR dyspareunia OR exhibitionism OR "orgasmic disorder" OR fetishism OR frotteurism OR "gender identity disorder" OR "impulse-control disorder" OR "intermittent explosive disorder" OR "erectile disorder" OR "nightmare" OR paraphilia OR pedophilia OR paedophilia OR "premature ejaculation" OR pyromania OR "sexual aversion disorder" OR "sexual masochism" OR "sexual sadism" OR sadomasochism OR "sexual maturation disorder" OR "transvestic fetishism" OR "egodystonic sexual orientation" OR "sexual relationship disorder" OR "sexual arousal disorder" OR dyspareunia OR trichotillomania OR vaginismus OR voyeurism OR suicid* OR self injur* OR "self harm" OR violen* OR offend* OR "Factitious disorder" OR munchausen OR "ganser syndrome"

***Search: in title abstract keywords* in Cochrane Reviews (Reviews only) and Trials (Word variations have been searched)**

148008

#11

#9 AND #10

915
