# Supplementary material for: Online Peer-to-Peer Support for Young People With Mental Health Problems: A Systematic Review
Source: JMIR Ment Health. 2015 May 19;2(2):e19. doi: 10.2196/mental.4418 (PMC4607385; doi:10.2196/mental.4418)
Supplement: Multimedia Appendix 3 [file mental_v2i2e19_app3.pdf]

Multimedia Appendix 3. Full data from the review.

**Study**<br><br>**Country**	**Participants**<br><br>**Symptom level at recruitment**<br><br>**Number of recruited participants**	**Age (Mean, SD)**<br><br>**Sex (% Female)**	**Intervention description**	**Peer support format**<br><br>**Peer support type**<br><br>**Moderation status**<br><br>**Moderator**	**Design**<br><br>**ITT**	**Completer (N)**<br><br>**Dropouts (n, %)**	**Primary outcome measures**<br><br>**Measurement points**	**Results for the effectiveness of online peer-to peer-support at post-intervention**	**Effect sizes and 95% confidence intervals (CI):**<br>**Hedges *g* (between groups at post-intervention)**
**Depression and anxiety**									
Ellis et al. (2011) [26]<br><br>Australia	Undergraduate students<br><br>"Elevated" psychological distress (low to moderate) measured with the K-10. Scores > 30 excluded.<br><br>N = 39<br><br>I^1 = 13<br>$I^{2\ peer\ support}$ = 13<br>C = 13	M = 19.67<br>SD = 1.66<br><br>F = 77%	I^1: "MoodGYM" (3 x weekly 1 hour sessions of online CBT, 5 modules plus therapist guidance)<br><br>$I^{2\ peer\ support}$: "Mood Garden" (3 x weekly 1 hour sessions of online peer-based support and information plus therapist guidance)<br><br>C: No intervention	Message boards - Forums with blogs and mood chart<br><br>Public<br><br>Moderated<br><br>Volunteers who experienced mood disorders	RCT<br><br>Yes	N = 39<br><br>No dropout	DASS (Depression)<br><br>DASS (Anxiety)<br><br>Baseline<br>Post intervention (3 weeks)	Depression:<br>Online peer support was not effective compared to the control condition.<br><br>Anxiety:<br>Online peer support was effective compared to the control condition.*	Depression:<br>I^2 vs C = -.63<br>CI: -1.41, 0.16<br><br>Anxiety:<br>I^2 vs C = -.91<br>CI: -1.72, 0.10
Horgan et al. (2013) [28]<br><br>Ireland	University students<br><br>Depressive symptoms measured with the CED-D (score>16)<br><br>N=118	M = 20.6<br>SD = 1.8<br><br>F = 35.6%	$I^{\ peer\ support}$: Web site provided a forum to allow participants to offer peer support to each other. Information on depression and links to other supports	Forum<br><br>Research<br><br>Moderated<br><br>Researcher	Pre-post<br><br>No	N = 16<br><br>n = 102 (86%)	CES-D<br><br>Baseline<br>Post intervention (6 weeks)	A non-significant reduction from pre to post-intervention.	N/A
**Psychological problems**									

Freeman et al. (2008) [27]<br><br>England	College students<br><br>Psychological problems<br><br>N = 283<br><br>I^{1} = 141<br>$I^{2 \text{ peer support}}$ = 142	M = 21<br>SD = not reported<br><br>F = 70%	I^{1}: Website containing information about common student problems<br><br>$I^{2 \text{ peer support}}$: Website with the addition of an online mutual support group which used an electronic bulletin board	Electronic bulletin board<br><br>Research<br><br>Unknown<br><br>Unknown	RT<br><br>No	I^{1} = 82<br>$I^{2 \text{ peer support}}$ = 51<br><br>I^{1} = 59 (42%)<br>$I^{2 \text{ peer support}}$ = 91 (64%)	CORE-OM<br><br>Baseline<br>Post intervention (10 weeks)	Adding online peer support did not increase the effectiveness of the intervention.	I^{1} vs I^{2} = -.22<br>CI: -0.57, 0.13
**Eating disorders**									
Low et al. (2006) [24]<br><br>USA	Undergraduate women<br><br>Non-symptomatic<br><br>N=61<br><br>I^{1} = 14<br>$I^{2 \text{ peer support}}$ = 19<br>$I^{3 \text{ peer support}}$ = 14<br>C = 14	M = not reported<br>SD = not reported<br><br>F = 100%	I^{1}: "Student Bodies" (8 weeks web-based computer program with no discussion group)<br><br>$I^{2 \text{ peer support}}$: "Student Bodies" (8 weeks web-based computer program with an unmoderated discussion group)<br><br>$I^{3 \text{ peer support}}$: "Student Bodies" (8 weeks web-based computer program with a clinically moderated discussion group)<br><br>C: No intervention	Discussion groups<br><br>Research<br><br>Unmoderated and moderated<br><br>Clinical psychologist	RCT<br><br>Yes, however, only some outcomes were subject to ITT analysis. Results below pertain to the completer analyses.	N = 61<br><br>n = 11 (15%)	EDI – Bulimia, Body Dissatisfaction, and Drive for Thinness subscales<br><br>Baseline<br>Post intervention (8 weeks)<br>Follow-up (11-12 months)	Bulimia:<br>Adding online peer support did not increase the effectiveness of the intervention.<br><br>Body Dissatisfaction:<br>Adding online peer support did not increase the effectiveness of the intervention.<br><br>Drive for Thinness:<br>Adding online peer support did not increase the effectiveness of the intervention.	Bulimia:<br>I^{1} vs I^{2} = .52<br>CI: 0.18, 1.22<br>I^{1} vs I^{3} = .98<br>CI: 0.20, 1.77<br><br>Body Dissatisfaction:<br>I^{1} vs I^{2} = -1.05<br>CI: -1.84, -0.26<br>I^{1} vs I^{3} = .07<br>CI: -0.67, 0.81<br><br>Drive for Thinness:<br>I^{1} vs I^{2} = -.48<br>CI: -1.18, 0.22<br>I^{1} vs I^{3} = -.59<br>CI: -1.34, 0.17
**Substance abuse (tobacco)**									
Woodruff et al. (2001) [29]	Rural teens<br><br>Smokers	M = 15<br>SD = not reported	$I^{\text{peer support}}$: Seven 1-hour intervention chat sessions in real-time virtual	Virtual world chat room<br><br>Research	Pre-post<br><br>No	N = 18<br><br>n = 8 (31%)	Percentage of smoking abstinence in the past week	A non-significant increase in the percentage of smoking abstinence	N/A

USA	N = 26	F = not reported	world with a trained cessation counsellor over a 2-month period	Moderated<br><br>Trained cessation counsellor			Baseline<br>Post intervention (2 months)<br>Follow-up (1 month)	from pre to post-intervention.	
Woodruff et al. (2007) [25]<br><br>USA	Adolescent smokers<br><br>Smokers<br><br>N = 136<br>I[1 peer support] = 77<br>C = 59	M = 16<br>SD = not reported<br><br>F = 46%	I[peer support]: Seven 45-minute intervention chat sessions in real-time virtual world with a trained cessation counsellor over a 7-week period<br><br>C: No intervention	Virtual world chat room<br><br>Research<br><br>Moderated<br><br>Trained cessation counsellor	RCT<br><br>Yes	N = 102<br><br>n = 34 (25%)	Past-week smoking abstinence (yes vs. no)<br><br>Baseline<br>Post-intervention (7 weeks)<br>Follow-up (3 months)<br>Follow-up (12 months)	Online peer support was effective compared to the control condition.*	N/A

Notes: I = intervention group, C = control group, RCT = randomized controlled trial, RT = randomized trial, CBT = cognitive behavioral therapy
Measures: DASS = Depression Anxiety Stress Scale, CES-D = Center for Epidemiologic Studies Depression Scale, EDI = The Eating Disorders Inventory, CORE-OM = The Clinical Outcomes in Routine Evaluation – Outcome Measure, K-10 = Kessler Psychological Distress Scale
* Significant difference between the online peer support intervention and the control group at post-intervention
